# A Hypomethylating Ketogenic Diet in Apolipoprotein E-Deficient Mice: A Pilot Study on Vascular Effects and Specific Epigenetic Changes

**DOI:** 10.3390/nu13103576

**Published:** 2021-10-13

**Authors:** Rita Castro, Courtney A. Whalen, Sean Gullette, Floyd J. Mattie, Cristina Florindo, Sandra G. Heil, Neil K. Huang, Thomas Neuberger, A. Catharine Ross

**Affiliations:** 1 Department of Nutritional Sciences, The Pennsylvania State University, University Park, PA 16802, USA; caw400@psu.edu (C.A.W.); fjm1311@gmail.com (F.J.M.); neil.huang@tufts.edu (N.K.H.); acr6@psu.edu (A.C.R.); 2 Faculty of Pharmacy, Universidade de Lisboa, 1649-003 Lisbon, Portugal; cristinaflorindo@ff.ulisboa.pt; 3 Research Institute for Medicines (iMed.ULisboa), Universidade de Lisboa, 1649-003 Lisbon, Portugal; 4 The Huck Institutes of the Life Sciences, The Pennsylvania State University, University Park, PA 16802, USA; sqg5746@psu.edu (S.G.); tun3@psu.edu (T.N.); 5 Medical Center Rotterdam, Department of Clinical Chemistry, Erasmus MC University, 3015 GD Rotterdam, The Netherlands; s.heil@erasmusmc.nl; 6 Jean Mayer USDA Human Nutrition Research Center on Aging, Cardiovascular Nutrition Laboratory, Tufts University, Boston, MA 02111, USA; 7 Biomedical Engineering, The Pennsylvania State University, University Park, PA 16802, USA

**Keywords:** endothelial dysfunction, 14T-MRI, H3K27me3, H3K27ac, histone methylation, histone acetylation, mild hyperhomocysteinemia, homocysteine and vascular disease

## Abstract

Hyperhomocysteneinemia (HHcy) is common in the general population and is a risk factor for atherosclerosis by mechanisms that are still elusive. A hypomethylated status of epigenetically relevant targets may contribute to the vascular toxicity associated with HHcy. Ketogenic diets (KD) are diets with a severely restricted amount of carbohydrates that are being widely used, mainly for weight-loss purposes. However, studies associating nutritional ketosis and HHcy are lacking. This pilot study investigates the effects of mild HHcy induced by nutritional manipulation of the methionine metabolism in the absence of dietary carbohydrates on disease progression and specific epigenetic changes in the apolipoprotein-E deficient (*apoE*^–/–^) mouse model. *ApoE*^–/–^ mice were either fed a KD, a diet with the same macronutrient composition but low in methyl donors (low methyl KD, LMKD), or control diet. After 4, 8 or 12 weeks plasma was collected for the quantification of: (1) nutritional ketosis, (i.e., the ketone body beta-hydroxybutyrate using a colorimetric assay); (2) homocysteine by HPLC; (3) the methylating potential S-adenosylmethionine to S-adenosylhomocysteine ratio (AdoHcy/AdoMet) by LC-MS/MS; and (4) the inflammatory cytokine monocyte chemoattractant protein 1 (MCP1) by ELISA. After 12 weeks, aortas were collected to assess: (1) the vascular AdoHcy/AdoMet ratio; (2) the volume of atherosclerotic lesions by high-field magnetic resonance imaging (14T-MRI); and (3) the content of specific epigenetic tags (H3K27me3 and H3K27ac) by immunofluorescence. The results confirmed the presence of nutritional ketosis in KD and LMKD mice but not in the control mice. As expected, mild HHcy was only detected in the LMKD-fed mice. Significantly decreased MCP1 plasma levels and plaque burden were observed in control mice versus the other two groups, together with an increased content of one of the investigated epigenetic tags (H3K27me3) but not of the other (H3K27ac). Moreover, we are unable to detect any significant differences at the *p* < 0.05 level for MCP1 plasma levels, vascular AdoMet:AdoHcy ratio levels, plaque burden, and specific epigenetic content between the latter two groups. Nevertheless, the systemic methylating index was significantly decreased in LMKD mice versus the other two groups, reinforcing the possibility that the levels of accumulated homocysteine were insufficient to affect vascular transmethylation reactions. Further studies addressing nutritional ketosis in the presence of mild HHcy should use a higher number of animals and are warranted to confirm these preliminary observations.

## 1. Introduction

Mild hyperhomocysteinemia (HHcy), a condition defined by an accumulation of plasma homocysteine (Hcy) between 15 and 25 μM, is highly prevalent in most Western populations and is considered an independent risk factor for atherosclerosis and cardiovascular disease (CVD) [1,2]. Nevertheless, the molecular basis of this association is poorly understood. Homocysteine is a sulfur-containing amino acid formed during methionine metabolism that may indirectly modulate cellular transmethylations, via its metabolic precursor, S-adenosylhomocysteine (AdoHcy) [2,3,4,5,6]. 

S-Adenosylmethionine (AdoMet) is the universal methyl donor compound formed from methionine. AdoHcy is formed upon the transfer of a methyl group from AdoMet to different substrates through the action of specific methyltransferases. Interestingly, excess AdoHcy may negatively regulate the activity of these AdoMet-dependent methyltransferases. Consequently, the ratio of AdoMet to AdoHcy (AdoMet:AdoHcy) is used as an index of cell methylating potential. AdoHcy is further hydrolyzed to Hcy by a reversible reaction that thermodynamically favors the production of AdoHcy rather than that of Hcy. Thus, when Hcy accumulates, AdoHcy may accumulate as well, decreasing the AdoMet/AdoHcy ratio, which may, in turn, decrease the methylation of epigenetically relevant targets, such as the nuclear proteins and histones [6,7,8,9]. For example, a reduction in the vascular content of the repressive epigenetic tag, tri-methylation of histone H3 at lysine 27 (H3K27me3), may favor atherosclerosis progression under HHcy [9]. Enhancer of zeste homolog 2 (EZH2) is the AdoMet-dependent histone methyltransferase that establishes the H3K27me3 epigenetic mark. EZH2 was identified as a new target affected by an endothelial hypomethylated status, which decreased the content of the epigenetic tag H3K27me3, promoting a pro-atherogenic phenotype [9]. However, the magnitude of the accumulated Hcy concentration and its repercussions on the cell methylation index (AdoMet:AdoHcy) may dictate their effect on vascular homeostasis and underlying epigenetic alterations. Studies with animal models of human atherosclerosis, in which the average plasma Hcy exceeded 25 μM, have confirmed the vascular toxicity of Hcy due to epigenetic dysregulation [2,4,10]. However, the findings associated with mild HHcy are inconsistent [4,7,10]. 

Diets with very low levels of carbohydrates, or ketogenic diets (KD), are widely used for weight-loss [11]. KD contain 70–80% of kcal from fat, which causes a shift in metabolism away from carbohydrates and towards the fatty acid oxidation, stimulating endogenous production of ketone bodies. β-Hydroxybutyrate (BHB) is the predominant ketone body that replaces glucose as the primary source of energy. BHB has been suggested as a histone deacetylase (HDAC) inhibitor that alters the epigenetic landscape [12]. However, the effect of KD-induced mild HHcy on plaque formation and vascular epigenetic tags remains unknown. Previous studies have addressed the atherogenic impact of mild Hcy and the underlying epigenetic deregulation in the absence of nutritional ketosis [7]. These include our recent study, in which we used a hypomethylating high-fat diet, but in which the presence of dietary carbohydrates precluded the establishment of nutritional ketosis [13]. The present research innovates by investigating the vascular effects of a hypomethylating high-fat diet, in which a markedly different dietary macronutrient profile, i.e., the absence of carbohydrates, triggers a stage of ketosis. Here, we used apolipoprotein E deficient (*apoE*^–/–^) mice, a model of atherosclerosis [13], to investigate the impact of this hypomethylating KD on the systemic levels of BHB plasma levels, inflammation, and methylating index AdoMet/AdoHcy. Moreover, we quantified the volume of aortic plaque and determined the vascular methylation index and its content of specific epigenetic tags.

## 2. Results and Discussion

Seven-week-old male *apoE*^–/–^ mice were fed a KD (Kcal %: 1/81/18, CHO (carbohydrate)/fat/protein), a diet with the same macronutrient profile but with a lower content in methyl donors (LMKD), or a control diet (Kcal %: 71/11/18, CHO/fat/protein) for 12 weeks. 

### 2.1. Body Weight

**Figure 1:**
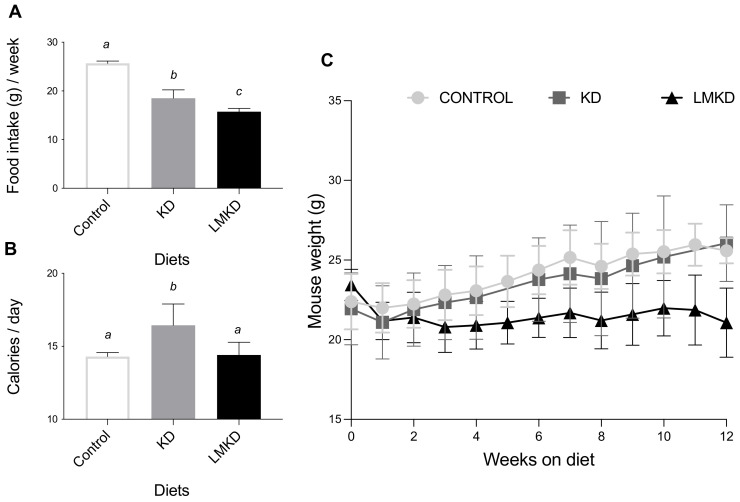
The effect of the experimental diets (ketogenic diet, KD; low methyl ketogenic diet, LMKD; and control diet) on food intake (**A**), calorie consumption (**B**) and animal growth (**C**). Data shown are the mean ± SD, *n* = 4–6/group. Means not sharing superscript letters differ by *p* < 0.05.

Mice fed the LMKD consumed significantly less food than the KD mice (Figure 1A). Moreover, the KD mice consumed significantly less food than the controls (Figure 1A). Nevertheless, because of the different caloric densities of the diets, KD mice consumed more calories than the LMKD and control groups (Figure 1B). Calorie intake did not differ in the two latter groups. Concerning the effect of the experimental diets on body weight, the results showed that mice fed a KD failed to gain weight compared to the controls, despite the higher fat content of the diet. Mice fed a KD transiently lost weight but then stabilized at a weight similar to that of the mice fed the control diet (Figure 1C). This anti-obesogenic effect of the KD, despite its high dietary fat content, has been reported in other studies using rodents [14,15]. 

Moreover, mice in the LMKD group were lighter than the other two groups of animals (Figure 1C), thus showing that these animals grew more slowly than KD- or control-fed mice. This observation emphasizes the essential role of those micronutrients for proper growth [16]. In fact, the LMKD was formulated to induce mild HHcy by decreasing the levels of vitamins B6, B9 and B12 in the presence of excess methionine. Previous studies [10,13,17] have also reported a significant decrease in the body weight of animals fed vitamin-B deficient diets. Thus, the very high fat content in the LMKD used here did not counterbalance the deleterious effect on the growth of a dietary treatment with suboptimal levels of micronutrients.

### 2.2. Nutritional Ketosis 

**Figure 2:**
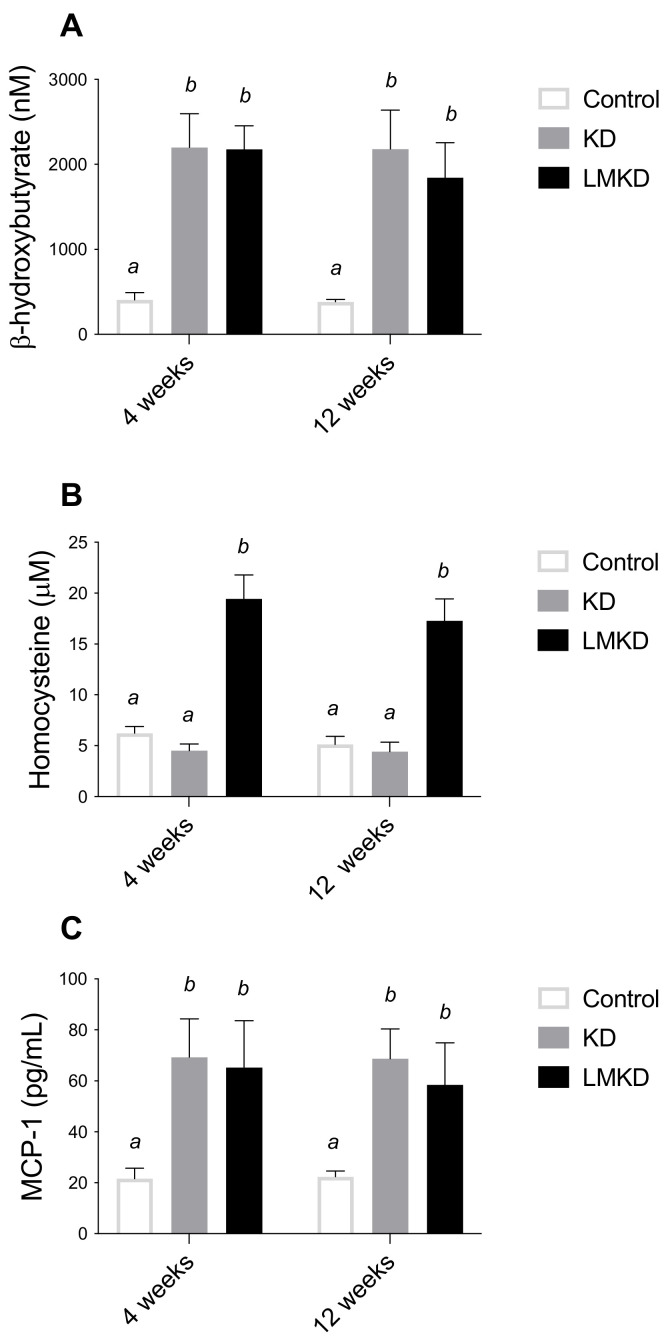
The effect of the experimental diets (ketogenic diet, KD; low methyl ketogenic diet, LMKD; and control) on the plasmatic concentrations of β-Hydroxybutyrate (**A**), homocysteine (**B**) and monocyte chemoattractant protein-1 (MCP-1) (**C**). Data shown are the mean ± SD, *n* = 4–6/group. Means not sharing superscript letters differ by *p* < 0.05.

Circulating BHB levels were measured after 4 and 12 weeks in each diet group (Figure 2A). At both time-points, blood BHB concentrations were similar within the same group of mice but significantly elevated in the KD- and LMKD-fed mice, compared to controls, confirming that a sustained nutritional ketosis was attained with both low carbohydrate diets (KD and LMKD). Taking both time-points together, KD- and LMKD-fed mice had significantly higher BHB concentrations (mean ± SD, nM) (2191 ± 370 and 2010 ± 346, respectively), compared to the control-fed mice (394 ± 50). The range of the BHB concentration was similar to other reports [18,19], indicating the reliability of this mouse model in understanding the effect of this ketone body on cardiometabolic diseases.

Interestingly, the levels of BHB in the KD and LMKD mice were always similar to each other (Figure 2A), thus showing that the inadequate levels of micronutrients in the LMKD did not affect the production of BHB, and thus a similar state of nutritional ketosis was attained in both mice groups (KD and LMKD). 

### 2.3. Plasma Homocysteine

A mild HHcy was effectively induced by the LMKD. In fact, after 4 and 12 weeks of exposure to this diet, mice had significantly higher plasma concentrations of total Hcy (tHcy) than KD- or control-fed mice (Figure 2B). The tHcy is defined as the total concentration of Hcy after the reductive cleavage of all disulfide bonds. The remethylation of Hcy to methionine requires folate and vitamin B12 (cobalamin) as co-factors, or betaine, a choline metabolite, as a methyl donor compound. Moreover, the catabolism of Hcy to cysteine requires vitamin B6. Thus, dietary manipulation of the content of B vitamins and choline is a well-established approach to produce the accumulation of Hcy in mice, especially in the presence of excess methionine [10,17,20]. The magnitude of the accumulated Hcy in the present study was similar to that observed previously [13], in which the same nutritional modulation was applied to diets having a higher carbohydrate content. Thus, the lower amount of carbohydrates in the diet of this study did not appear to modify the effect of suboptimal levels of micronutrients involved in methionine metabolism on tHcy levels in circulation. 

### 2.4. Plasma MCP-1 Levels 

At the same time, i.e., after 4 and 12 weeks on each diet, the plasma levels of the monocyte chemoattractant protein 1 (MCP-1) were determined. MCP-1 is an inflammatory chemokine previously identified as an essential mediator in the pathogenesis of atherosclerosis in humans and *apoE*^–/–^ mice [21,22]. Specifically, MCP-1 is involved in the formation, progression and destabilization of atheromatous plaques [4,22]. Our results show that plasma MCP-1 concentrations were significantly higher in mice fed either the KD or LMKD than in control mice (Figure 2C), suggesting a pro-inflammatory effect of both high-fat low-carbohydrates diets, regardless of the different Hcy levels. This observation agrees with the positive effect of dietary fat on systemic inflammation that has been consistently reported in studies using diets containing a lower proportion of carbohydrates [23]. Moreover, we were unable to detect any significant differences at the *p* < 0.05 level for MCP1 plasma levels between KD or LMKD mice. Thus, despite a significant increase in plasma tHcy concentrations in the LMKD than in KD mice, the maintenance of the MCP1 concentrations suggests that both groups showed comparable levels of systemic inflammation. Nevertheless, the significance of this observation is limited by the low number of animals used in this pilot study. Moreover, vascular MCP-1 levels were shown to be pathogenetically involved in atherogenesis in murine models of atherosclerosis [24]. However, here, we did not measure the concentrations of MCP-1 in the aortic tissue, thus, we recognize this limitation of the present study. We also recognize that, in our study, other cytokines could have been quantified in plasma to unequivocally address and compare the levels of systemic inflammation between the KD or LMKD groups. For example, interleukin 15 (IL-15) is a recently identified critical mediator of inflammation in the pathogenesis of CVD [25] that was shown to play a significant role in promoting atherosclerosis in *apoE*^–/–^ mice [26], and whose expression was disturbed when wild-type mice were fed KD [27]. 

### 2.5. Systemic and Vascular Methylation Indexes and Concentrations of Relevant Metabolites

**Figure 3:**
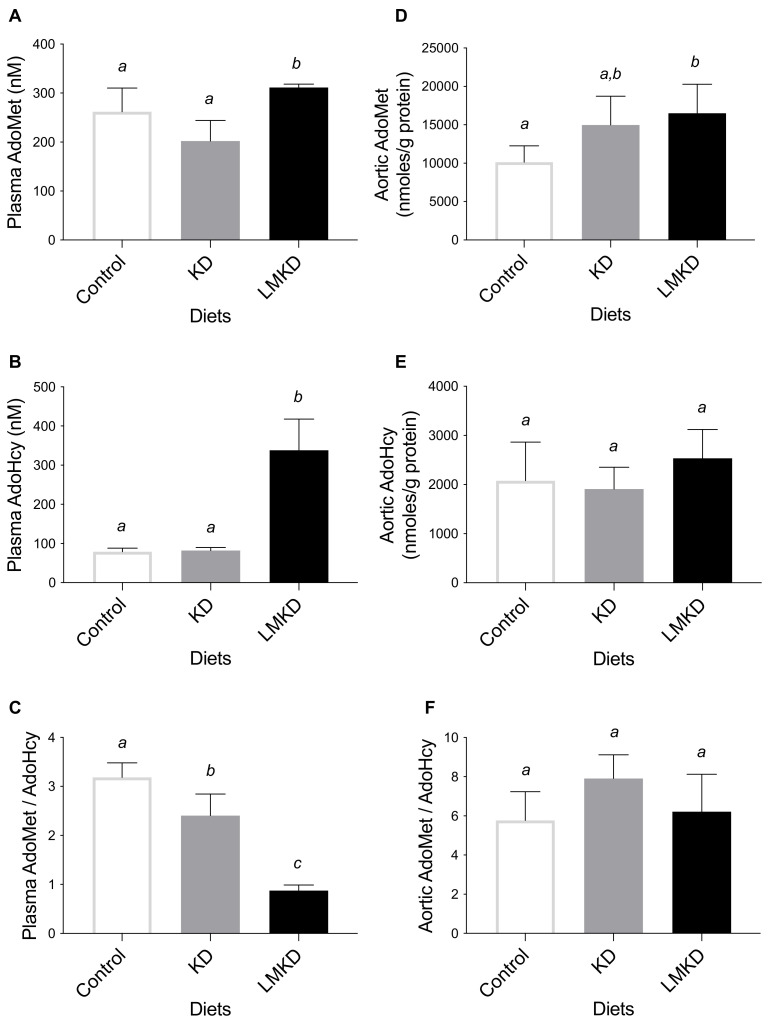
The effect of the experimental diets (ketogenic diet, KD; low methyl ketogenic diet, LMKD; and control) on the concentrations of plasma S-adenosylmethionine (AdoMet) (**A**), S-adenosylhomocysteine (AdoHcy) (**B**) and AdoMet/AdoHcy ratio (**C**), as well as of aortic AdoMet (**D**), AdoHcy (**E**) and AdoMet/AdoHcy ratio (**F**). Data shown are the mean ± SD, *n* = 4–6/group. Bars not sharing superscript letters differ by *p* < 0.05.

The ratio of AdoMet concentration to AdoHcy concentration was used as an indicator of the methylating capacity and determined in plasma and aortas. LMKD mice displayed a significantly lower plasma AdoMet:AdoHcy ratio, as well as a significantly higher plasma AdoMet and AdoHcy than in KD or control mice (Figure 3). Thus, even with the limited number of mice used in this study, a significant systemic hypomethylation was detected in the LMKD group versus the other two groups of mice. Moreover, a significantly decreased plasma AdoMet:AdoHcy ratio was detected in the mice fed the KD versus the controls, unraveling the presence of systemic hypomethylation in KD animals. This observation suggests the existence of an interaction between the level of dietary carbohydrates and the systemic methylation index that deserves further investigation. Interestingly, however, the aortic concentrations of AdoMet, AdoHcy, and AdoMet/AdoHcy were mostly similar in all groups of mice, suggesting a similar vascular methylating environment in all groups, despite the observed differences in the systemic methylation status. When Hcy accumulates intracellularly, AdoHcy may accumulate as well [6]. Thus, we argue that the vascular levels of accumulated Hcy were insufficient to promote AdoHcy build-up and thus aortic hypomethylation, a possibility that still needs to be confirmed in future studies. In fact, the methylation regulating enzymes in murines are differentially expressed, causing tissue-specific differences in AdoMet and AdoHcy concentrations [20,28,29]. 

### 2.6. Atherosclerotic Plaque Burden

**Figure 4:**
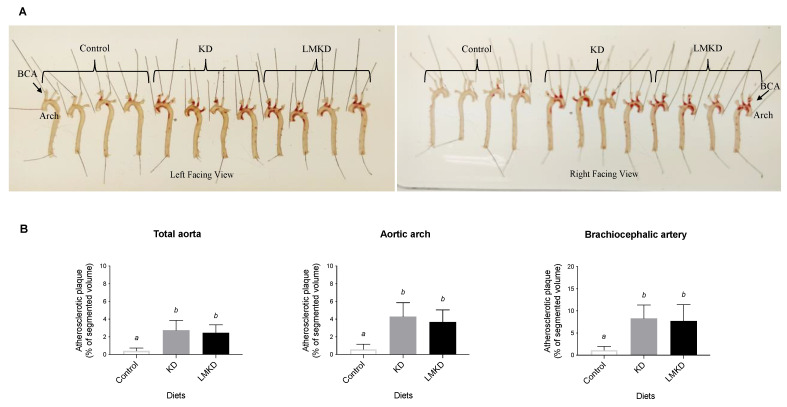
The effect of the experimental diets (ketogenic diet, KD; low methyl ketogenic diet, LMKD; and control) on aortic plaque burden. (**A**) Representative images of aortas from mice fed the experimental diets, after lipid staining (in red) with Oil Red-O (both sides of the aortas are shown), showing the localization of the aortic arch and brachiocephalic artery (BCA). (**B**) Volumetric assessment of plaque burden in different aortic segments by 14T-MRI. Data shown are the mean ± SD, *n* = 4–6/group. Bars bearing different letters were significantly different (*p* < 0.05).

Two different methods evaluated the effect of each diet on atherosclerosis progression. First, classical Oil Red-O staining was used to estimate the amount of lipid-rich plaques in each aorta [13]. As shown in Figure 4A, the results suggested a similar amount of plaque in KD and LMKD aortas, which was significantly higher than in the controls. These observations were confirmed by determining the volume of the aortic atheroma using magnetic resonance imaging (MRI). The results showed that control animals presented significantly less aortic atherosclerosis burden than KD or LMKD mice, with no significant differences detected between the latter two groups at the *p* < 0.05 level (Figure 4B). The atherosclerotic lesions were distributed in the same aortic regions (aortic arch and brachiocephalic artery) in all three groups. Therefore, the differences between KD or LMKD aortas and control aortas in these two highly susceptible regions, the aortic arch and brachiocephalic artery, were even more pronounced (Figure 4B). However, consistent with the similar systemic levels of MCP-1 in the KD and LMKD groups (Figure 2C), the volume of aortic atheroma did not differ in these groups (Figure 4B). Nevertheless, the significance of this observation is limited by the number of animals used in this pilot study. Furthermore, the deleterious effect on cell growth caused by the inadequacy of several micronutrients in the LMKD may have retarded the plaque development, contributing to the similar plaque burden observed in the KD and LMKD groups. Other studies in *apoE*^–/–^ mice [13,30], but not all [31], also reported this absence of vascular toxicity associated with mild HHcy. 

Lastly, previous studies using murine models that mimic human atherosclerosis have also reported the proatherogenic effect of low-carbohydrate diets, although they have not assessed nutritional ketosis in these models. For example, Foo et al. [32] observed a significant acceleration of atherosclerosis in *apoE*^–/–^ mice fed a low-carbohydrate high-protein diet compared to control mice. Kostogrys et al. used a double knockout mouse model of experimental atherosclerosis, *apoE*^–/–^/*LDLR*^–/–^, which also lacks the low-density lipoprotein (LDL) receptor, and reported the atherogenic effect of a low-carbohydrate diet, when compared to a control diet, in two different studies [33,34]. 

### 2.7. Specific Histone H3 Post-Translational Modifications at Lysine 27: H3K27me3 and H3K27ac

A reduction in the vascular content of the repressive epigenetic tag, H3K27me3, may contribute to the atherosclerosis progression under HHcy [2,4,7]. In previous cell studies, we documented the ability of a hypomethylating environment to decrease the endothelial H3K27me3 promoting an atherogenic phenotype [9]. Nevertheless, this observation was not reproduced in a recent in vivo study, suggesting that, in this study model (*apoE*^–/–^ mice) and under conditions of mild HHcy, systemic hypomethylation is insufficient to promote vascular hypomethylation and decrease histone H3 methylation at the K27 level [13]. 

Recent studies suggest that BHB is an endogenous inhibitor of histone deacetylases that may impact gene expression [35]. In fact, ketosis has been linked to covalent histone modifications, which serve as dynamic regulators of chromatin architecture and gene transcription [36]. These include the lysine residue 27 on histone H3 that can be subjected to methylation or acetylation, forming the epigenetic tags H3K27me3 or H3K27ac, respectively, which have opposing effects on chromatin structure [12]. Moreover, a crosstalk between these two epigenetic tags was reported, reporting that the loss of the tag H3K27me3 results in a reciprocal gain of the tag H3K27ac [12]. These observations lead us to focus, in addition to the H3K27me3 repressive mark, on the H3K27ac permissive epigenetic tag. 

**Figure 5:**
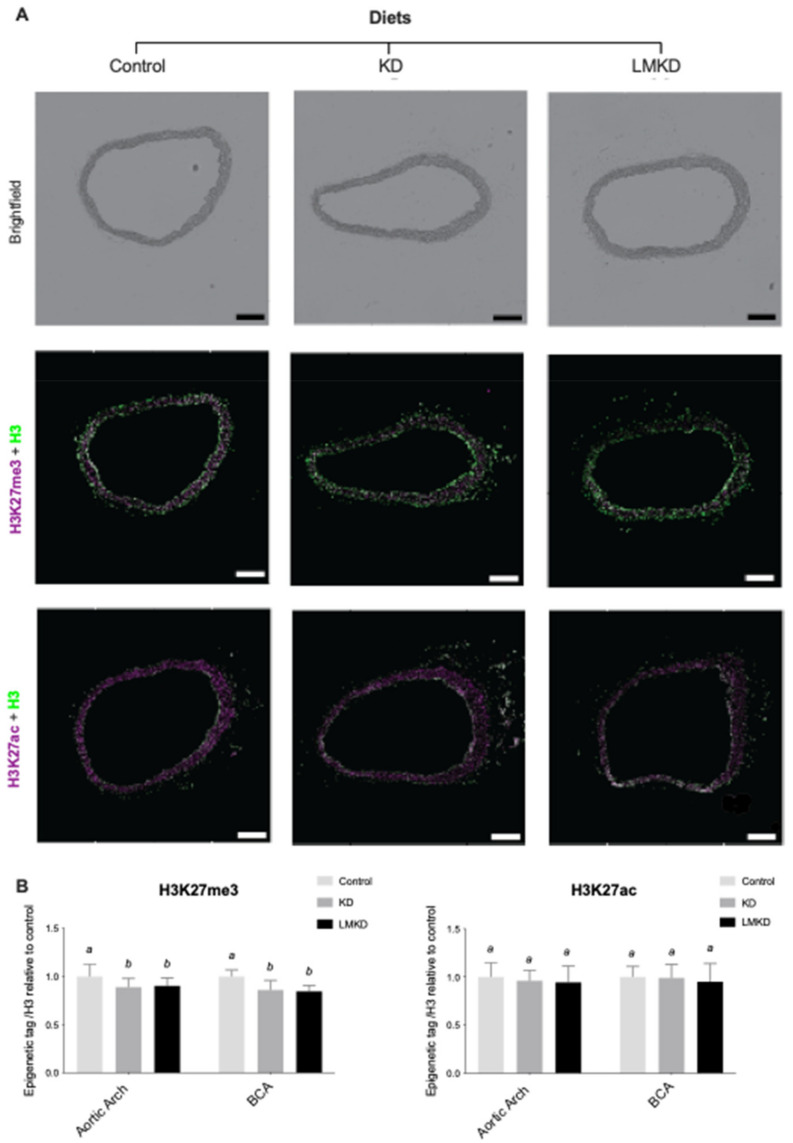
The effect of the experimental diets (ketogenic diet, KD; low methyl ketogenic diet, LMKD; and control) on the vascular content of specific epigenetic tags at lysine 27 on histone H3 (H2K27me3, tri-methylation; H3K27ac, acetylation) in the aortic arch and brachiocephalic artery (BCA). (**A**) Representative images of brightfield and immunofluorescence quantification of BCA cross sections. Total histone 3 (H3) in green, and histone 3 trimethylation at lysine 27 (H3K27me3) or acetylation (H3K27ac) in magenta. Scale bars = 200 μm. (**B**) Immunohistochemistry results. Data are the mean ± SD, *n* = 35. Bars bearing different letters were significantly different (*p* < 0.05).

Thus, the effect of the experimental diets on the aortic content of H3K27me3 and H3K27ac was evaluated by immunofluorescence. Two different aortic segments, the aortic arch and the brachiocephalic artery, were considered. However, in none of the regions considered was any significant difference detected in the relative content of the two epigenetic marks in LMKD and KD aortas (Figure 5). 

These pilot results support our recent observations in the same animal model, but using diets with a higher carbohydrate content, in which mice exposed to mild Hcy accumulation (<25 μM) for 12 weeks showed similar levels of vascular of H3K27me3 and atheroma as the ones under normal plasma Hcy concentrations [13]. However, as opposed to our previous study, here the aortas from the control group were also used to quantify the specific epigenetic content. Interestingly, the results showed that in both the KD or LMKD aortas, the content of H3K27me3 was significantly decreased versus the controls (Figure 5). As discussed above, a profound difference in the aortic atherosclerosis burden was observed between the control aortas and KD or LMKD aortas but not between the latter two aortas (Figure 4). Thus, even with the limited numbers of animals used in this pilot study, we could observe significantly lower levels of the content of the epigenetic tag H3K27me3 in the aortas presenting a higher atherosclerotic burden, favoring the involvement of this epigenetic tag on the atherosclerotic process. In support of this, a reduction in H3K27me3 content with the progression of human atherosclerotic plaques has been reported [37,38]. In fact, it has been established that EZH2 target genes encode several mediators involved in the establishment and progression of endothelial activation, a key step in the establishment of atherosclerosis [39]. Lastly, in the present study, we were unable to detect any effect of nutritional ketosis on specific histone acetylation. In fact, the vascular content of the epigenetic tag H3K27ac was similar among KD, LMKD and control groups (Figure 5), despite profound differences in vascular atheroma (Figure 4). These observations suggest that vascular H3K27ac level is not associated in the progression of atherosclerosis, at least in this model (*apoE*^–/–^ mice). In fact, the aortic concentration of H3K27ac was independent of the H3K27me3 mark and systemic BHB concentration. Previous studies revealed that BHB induced histone hyperacetylation in the in vitro and in vivo models [19,40,41,42]; however, more recent data did not confirm the function of BHB as a histone deacetylase inhibitor [35]. Thus, the role of BHB on histone acetylation remains unclear, and additional studies are required to elucidate this mechanism.

## 3. Materials and Methods 

### 3.1. Animals and Diets

Seven-week-old *apoE*^–/–^ mice, purchased from Jackson Laboratory (Bar Harbor, ME, USA) were housed individually in stainless steel wire-bottom cages in a temperature- and humidity-controlled room. Only male mice were used to control for the known effect of gender on atherosclerosis in this strain [43]. The animals had free access to water and to one of the following diets prepared based on AIN 93M (Research Diets, New Brunswick, NJ, USA): a control diet (11 Kcal% fat, 70 Kcal% carbohydrate, 18 Kcal% protein), a KD diet (81 Kcal% fat, 1 Kcal% carbohydrate, 18 Kcal% protein and 0.15% cholesterol), or an LMKD diet (80 Kcal% fat, 1 Kcal% carbohydrate, 19 Kcal% protein and 0.15% cholesterol) that was enriched in methionine and had reduced levels of methyl donors and vitamins (folate, choline, vitamin B6 and vitamin B12), as previously described in detail [13]. The macronutrient composition of the experimental diets is shown in Table 1. Diets were replaced once a week, at which time animals and the remaining food were weighed to determine food consumption and body weight progression. All procedures were performed in compliance with the Institutional Animal Care and Use Committee of the Pennsylvania State University, which specifically approved this study. 

### 3.2. Blood Collection 

After mice were fed for 4, 8 and 12 weeks, blood was collected from the retro-orbital cavity into heparinized tubes and immediately placed on ice. Plasma was isolated by centrifugation at 4 °C and immediately stored at −80 °C prior to further biochemical analyses.

### 3.3. Aorta Collection and Preparation of Aorta Lysates

After 12 weeks, mice were euthanized by carbon dioxide inhalation and aortas were collected, as previously described in detail [13]. Briefly, aortas were perfused with 10 mL of cold phosphate saline buffer (PBS). Approximately one-third of each aorta was excised, transferred into 100 µL of a solution of acetonitrile, methanol and water (2:2:1) containing 0.1M HCl, immediately immersed into liquid nitrogen and stored at −80 °C until the preparation of the aorta lysates described below. The remainder of each aorta, after perfusion with 10% neutral buffered formalin (NBF) in PBS, was fixed in 10% NBF overnight and processed, as described in 3.5.1. 

Aorta lysates were prepared by homogenization with 2.8 mm ceramic beads using a Bead Ruptor 12 (Omni, Kennesaw, GA, USA) with two cycles of 15 s at the maximum speed, with 30 s on ice between the cycles. The lysate was cleared by centrifugation at 12,000× *g* and 4 °C for 10 min and immediately stored at −80 °C until AdoHcy and AdoMet analyses were performed.

### 3.4. Biochemical Analyses

#### 3.4.1. Nutritional Ketosis

Plasma was tested for content of BHB using a colorimetric assay kit (Cayman Chemical, Ann Arbor, MI, USA) following the manufacturer’s instructions. 

#### 3.4.2. Homocysteine 

Plasma tHcy was determined by a high-performance liquid chromatography (HPLC) analysis adapted from the method of Araki and Sako [44].

#### 3.4.3. Systemic Inflammation

MCP-1 was measured in duplicate using plasma collected at 4 weeks and 12 weeks, by ELISA (Meso Scale Diagnostics, Rockville, MD, USA) following the manufacturer’s instructions.

#### 3.4.4. Plasma and Vascular Methylating Index

AdoMet and AdoHcy levels were measured in plasma and in aortic lysates using a liquid chromatography tandem-mass spectrometry (LC-MS/MS) method, after using solid-phase extraction columns according to Gellekink et al. [45,46]. The stability of AdoMet and AdoHcy in plasma was shown to be 100% if samples were stored immediately at −80 °C [46]. To protect AdoMet in aortic lysates from degradation, 0.1M HCl was used to acidify the aortic lysates, after which samples were stored at −80 °C [45]. 

### 3.5. Aorta Processing and Assessment of Vascular Lesions

Fixed aortas were stained with Oil Red-O (EMD Millipore Corporation, Billerica, MA, USA) to visualize adventitial fat removal during dissection [47]. Briefly, aortas were dehydrated in successive methanol–water solutions (25%, 50% and 78% methanol for 15 min each at room temperature, RT, with rocking). Aortas were then incubated in an Oil Red-O working solution (5 parts 2.8% Oil Red-O in methanol: 2 parts 1 M sodium hydroxide) for 2h at RT, with rocking. Subsequently, aortas were washed and rehydrated with successive PBS solutions with the following methanol concentrations (%): 78, 50, 25 or 0 (15 min each solution, RT, rocking). Connective tissues surrounding the aortas were dissected away under a stereomicroscope. The top of the heart attached, along with the aortic root, was removed. Complete staining of the atherosclerotic plaques with Oil Red-O was undertaken as previously described in detail [13,47]. Aortas were pinned out intact and photographed on each side.

#### 14T-MRI Analysis

Aortas were equilibrated in a solution of 0.1% Magnevist (Bayer, Whippany, NJ, USA), 0.25% sodium azide in PBS overnight at 4 °C, then placed into glass tubes (6 mm OD, 4 mm ID and 60 mm length) for MRI analysis. Aortic plaque burden was then quantified using an Agilent 14T micro imaging system, as previously described in detail [13,48]. A gradient echo imaging sequence with an imaging time of 9 h 48 min was used to generate 3D datasets of the aortas [49]. Scan parameters included an echo time (TE) of 13 ms, a repetition time (TR) of 100.00 ms, eight averages, a field of view (FOV) of 12.6 × 4.2 × 4.2 mm^3^, and a matrix size of 630 × 210 × 210, resulting in an isotropic resolution of 20 µm. After acquisition, MR data was reconstructed using Matlab (The MathWorks Inc., Natick, MA, USA). Zero-filling by a factor of 2 in each direction led to a final isotropic pixel resolution of 10 µm.

Data segmentation was performed using Avizo 9.5 (Thermo Fisher Scientific, Waltham, MA, USA). The lumen of the aorta, the different plaques and the aorta wall were manually segmented using the lasso tool. Quantification of plaque volume was determined using the material statistics function in Avizo 9.5 on the segmented aorta. The results were expressed as the percent of plaque area in relation to the total segmented area (plaque, lumen, and wall).

### 3.6. Aorta Immunofluorescence Analysis: Specific Histone Methylation and Acetylation

Following MRI analysis, aortas were equilibrated to 15%, then 30% sucrose solution was added as a cryoprotectant. Aortas were then further dissected to obtain arch and branch regions, grouped into arrays and embedded in Tissue-Tek^®^ Optimal Cutting Temperature compound (OCT, Sakura^®^ Finetek, Torrance, CA, USA). Twelve-µm thick tissue section arrays of each region were then mounted to TruBond™ 380 microscope slides, dried, and subjected to heat induced epitope retrieval (HIER) as previously described in detail [13]. Following HIER, tissue arrays were then encircled with an ImmEdge PAP pen (Vector Laboratories, Burlingame, CA, USA), blocked with 2% BSA, 1% NGS, 0.05% Triton X-100, 0.05% Tween-20 and 0.05% sodium azide in PBS (blocking buffer) and then incubated with primary antibodies in a blocking buffer overnight at 4°C. After washing, arrays were incubated in secondary antibody for 1 h at RT, washed, treated with Vector^®^ TrueVIEW^®^ Autofluorescence Quenching Kit (Vector Laboratories, Burlingame, CA, USA) and sealed under coverslips in VECTASHIELD^®^ Vibrance™ Antifade Mounting Medium (Vector Laboratories, Burlingame, CA, USA). The antibodies used were: rabbit anti-histone H3K27me3 pAb (1:200, Epigentek, Cat.# A-4039), rabbit anti-histone H3K27ac pAb (1:200, Epigentek, Cat.#A-4708), mouse anti-histone H3 mAb 1B1-B2 (1:200, BioLegend, cat.#819404), Alexa Fluor 555 goat anti-rabbit IgG (1:250, Invitrogen, Cat.# A-21428) and Alexa Fluor 488 goat anti-mouse IgG3 (1:250, Invitrogen, Cat.# A-21151). 

Immunofluorescence images were collected on an Olympus BX61 widefield fluorescence microscope at the Penn State Microscopy Facility (University Park, PA, USA). Images were segmented and quantified using FIJI [50], as previously described in detail [13]. Briefly, the aortic or arterial tissue were segmented manually into regions of interest (ROIs). Histone H3 fluorescence was used to define nuclei within the vessel and fluorescence within these nuclei was measured for both the histone H3 and histone H3K27me3 or H3K27ac immunofluorescence images. Data were then calculated as a ratio of H3K27me3:H3 fluorescence or H3K27ac:H3 fluorescence to assess vascular changes in the corresponding epigenetic tags.

### 3.7. Statistical Analyses

Analyses were performed in GraphPad Prism 7 (GraphPad Software, La Jolla, CA, USA), with statistical significance set to *p* < 0.05. For comparison of two groups, an unpaired Student’s *t*-test was used. For more than two groups, a one- or two-way analysis of variance (ANOVA) was performed with adjustment for multiple comparisons (Tukey’s multiple comparisons test).

## 4. Conclusions

In conclusion, a mild Hcy accumulation induced by a very low-carbohydrate diet containing low amounts of methyl donors promoted nutritional ketosis and systemic hypomethylation. Thus, the nutritional-induced manipulation of the Hcy metabolism was not affected by the BHB build-up in the LMKD group. However, we did not detect any significant differences, at the *p* < 0.05 level, in the volume of vascular lesions between the groups under nutritional ketosis (i.e., KD and LMKD). Nevertheless, the negative impact of the LMKD on cell growth, by retarding plaque progression, may have contributed to this outcome. We also did not detect a significant difference in the AdoMet:AdoHcy ratio or specific histone methylation between these two groups of animals. However, the significance of these observations is limited, not only by the small number of animals used, but also because we did not assess the methylation status of other potential targets, as in, for example, the protein arginine methylation status [20,28,29]. Interestingly however, we could observe lower levels of the content of the epigenetic tag H3K27me3 in the aortas presenting a higher atherosclerotic burden (KD and LMKD) versus the control, favoring the involvement of this epigenetic tag on the atherosclerotic process in this model. Lastly, the aortic concentration of the epigenetic tag H3K27ac was similar among the three groups of mice, albeit significant differences in vascular atheroma, showing that this epigenetic tag was independent of the H3K27me3 mark and the burden of atherosclerosis.

The results of the present pilot study are supported by our previous report, in which mild HHcy was induced in the same animal model with diets with a different macronutrient profile, without favoring atherosclerosis progression [13]. Thus, this absence of vascular toxicity associated with mild HHcy may be independent of the macronutrient profile of the diet, i.e., in the presence of nutritional ketosis. Furthermore, these intriguing observations suggest that, in both studies, the levels of accumulated Hcy were insufficient to disturb vascular homeostasis. However, this possibility needs to be taken cautiously due to the limited number of animals used in both studies. Nevertheless, and interestingly, evidence collected in human studies also suggests that the magnitude of tHcy build-up determines the correspondent vascular phenotype. In fact, lowering plasma tHcy in patients with severe HHcy drastically reduces the occurrence of adverse CVD events [5]. However, this same intervention in patients with mild HHcy will decrease CVD risk only if the baseline plasma tHcy is above a certain threshold [51]. 

As mentioned repletely above, one limitation of the current pilot study is the small sample size, as the analyses performed are all labor-intensive. However, a strength is that we have tested a mild form of HHcy, such as may be likely in humans [5]. Nevertheless, we acknowledge that confirmatory studies with a higher number of animals should confirm these pilot observations. Additional studies are being conducted to assess how a more profound accumulation of Hcy impacts vascular methylation status and atherosclerosis progression.

## Figures and Tables

**Table 1:** Acronutrient composition of the experimental diets (control, ketogenic diet, KD; and low methyl ketogenic diet, LMKD.

Macronutrient by gm (gm per 4000 Kcal Total)	Control	KD	LMKD
Casein	182	181	181
Corn Starch	435	0	0
Maltodextrin 10	156	0	0
Sucrose	101	0	0
Cellulose	35	35	35
Cocoa Butter	0	100	100
Primex (Non Trans-Fat)	25	233	232
Corn Oil	25	25	25

## Data Availability

The data presented in this study are available on request from the corresponding author.
